# Prognostic model based on the geriatric nutritional risk index and sarcopenia in patients with diffuse large B-cell lymphoma

**DOI:** 10.1186/s12885-020-06921-2

**Published:** 2020-05-18

**Authors:** Se-Il Go, Hoon-Gu Kim, Myoung Hee Kang, Sungwoo Park, Gyeong-Won Lee

**Affiliations:** 1 Division of Hematology-Oncology, Department of Internal Medicine, Gyeongsang National University Changwon Hospital, Gyeongsang National University College of Medicine, Changwon, Republic of Korea; 2 Institute of Health Science, Gyeongsang National University College of Medicine, Jinju, Republic of Korea; 3 Division of Hematology-Oncology, Department of Internal Medicine, Gyeongsang National University Hospital, Gyeongsang National University College of Medicine, Gangnam-ro 79, Jinju, 52727 Republic of Korea

**Keywords:** Lymphoma, large B-cell, diffuse, Serum albumin, Body weight, Cachexia, Sarcopenia

## Abstract

**Background:**

Systemic inflammation and cachexia are associated with adverse clinical outcomes in diffuse large B-cell lymphoma (DLBCL). The Geriatric Nutritional Risk Index (GNRI) is one of the main parameters used to assess these conditions, but its efficacy in DLBCL is inconclusive.

**Methods:**

We retrospectively reviewed 228 DLBCL patients who were treated with R-CHOP immunochemotherapy (rituximab plus cyclophosphamide, doxorubicin, vincristine, and prednisone). The patients were stratified according to GNRI score (> 98, 92 to 98, 82 to < 92, and < 82) as defined in previous studies. Additionally, the extent of sarcopenia was categorized as sarcopenia-both, sarcopenia-L3/PM alone, and non-sarcopenia-both according to skeletal muscle index.

**Results:**

Survival curves plotted against a combination of GNRI and sarcopenia scores revealed two clear groups as follows: high cachexia risk (HCR) group (GNRI < 82, sarcopenia-both, or GNRI 82–92 with sarcopenia-L3/PM alone) and low cachexia risk (LCR) group (others). The HCR group had a lower complete response rate (46.5% vs. 86.6%) and higher frequency of treatment-related mortality (19.7% vs. 3.8%) and early treatment discontinuation (43.7% vs. 8.3%) compared with the LCR group. The median progression-free survival (PFS) (not reached vs. 10.3 months, *p* <  0.001) and overall survival (OS) (not reached vs. 12.9 months, *p* <  0.001) were much shorter in the HCR group than in the LCR group. On multivariable analyses, the HCR group was shown to be an independent negative prognostic factor for PFS and OS after adjusting the National Comprehensive Cancer Network-International Prognostic Index (NCCN-IPI).

**Conclusions:**

A combined model of GNRI and sarcopenia may provide prognostic information independently of the NCCN-IPI in DLBCL.

## Background

Diffuse large B-cell lymphoma (DLBCL) is the most common subtype of adult non-Hodgkin lymphoma. Despite its aggressive nature, DLBCL is a potentially curable disease when treated with immunochemotherapy consisting of rituximab plus cyclophosphamide, doxorubicin, vincristine, and prednisone (R-CHOP) [1–3]. The International Prognostic Index (IPI) and its variations are well-known prognostic markers for DLBCL [4–6]; however, these indices remain limited in their ability to predict disease prognosis in disease such as this, where survival remains < 50%. Inability to predict clinical outcomes may be due, in part, to the heterogeneous nature of the disease, consisting of several molecular subtypes including germinal center B-cell-like (GCB) and activated B-cell-like (ABC) types [7]. Recently, five robust DLBCL subsets were detected using whole-exome sequencing. These subsets were shown to be a better predictor of disease prognosis relative to IPI scores [8]. Furthermore, comprehensive geriatric assessment could identify non-fit patients in whom curative intent treatment did not improve the prognosis [9]. Development of such novel prognosticators for disease outcomes remains a significant unmet need, allowing doctors to individualize treatment strategies for DLBCL patients.

Cancer cachexia is a multifactorial syndrome characterized by ongoing loss of skeletal muscle mass, malnutrition, and progressive functional impairment [10]. Cancer cachexia is associated with increased treatment-related toxicity and poor prognosis in cancer patients [11–13]. Given the high tumor burden of DLBCL and the favorable response rate with substantial treatment-related toxicities of R-CHOP treatment, the prognostic role of cancer cachexia is also likely to be observed in DLBCL patients. Several markers for malnutrition and cachexia such as body mass index (BMI), sarcopenia, adipopenia, and serum albumin level have been studied and suggested to be prognostic factors in DLBCL [14–17]. Additionally, the clinical value of the Geriatric Nutritional Risk Index (GNRI), which was originally developed to predict nutrition-related morbidity and mortality in non-cancer patients [18], was evaluated in two previous DLBCL studies with conflicting results [19, 20]. In this study, we re-evaluated the clinical impact of the GNRI on patient outcomes, both alone and in combination with sarcopenia.

## Methods

### Patients

All DLBCL patients (*n* = 262) treated with R-CHOP as first-line treatment between 2004 and 2017 at a single institution were retrospectively evaluated. The study was approved by the Institutional Review Board of Gyeongsang National University Hospital. Eligible patients were aged 18 years or older, had baseline CT scans for chest and abdomen, and had the records for height, body weight, and serum albumin level measured within a week before the beginning of R-CHOP (*n* = 246). Exclusion criteria were patients who had active infections (*n* = 7), double primary malignancy (*n* = 4), histologic transformation from low-grade lymphoma (*n* = 3), and lack of information for the National Comprehensive Cancer Network-International Prognostic Index (NCCN-IPI) at the time of measurement of GNRI and sarcopenia (*n* = 4). Finally, 228 patients were included in the analysis.

### Definitions of clinical variables

Pretreatment demographics and clinical variables were collected via electronic medical records. Body mass index (BMI) of less than 23.0 kg/m^2^ was classified to be underweight according to the Asian standard [21]. The response to R-CHOP along with any treatment-related toxicities were assessed using the revised International Working Group response criteria and the National Cancer Institute Common Toxicity Criteria (version 4.0). Relative dose intensity (RDI) was defined as the percentage of the actual total dose of each drug relative to the planned dose of the drug. Early treatment discontinuation was defined as any treatment prematurely terminated for reasons not due to disease progression. Treatment-related mortality was defined as any death not due to disease progression occurring within a month of the R-CHOP treatment or as death, at any time, that was apparently related to the R-CHOP treatment.

To determine the extent of sarcopenia, we measured muscle mass by CT histogram analysis, as described previously [16, 22]. Briefly, the muscle masses of the third lumbar level and of the pectoralis major and minor were measured and converted to L3 skeletal muscle index (L3-SMI) and pectoralis muscle SMI (PM-SMI), respectively, by dividing muscle mass by height in meters squared (cm^2^/m^2^). The patients were considered to be sarcopenic if their SMIs were lower than their respective cut-off values (L3-SMI, 52.4 cm^2^/m^2^ in males and 38.5 cm^2^/m^2^ in females; PM-SMI, 4.4 cm^2^/m^2^ in males and 3.1 cm^2^/m^2^ in females) [16, 22]. The extent of sarcopenia was defined as follows: non-sarcopenia-both, neither L3- nor PM-SMI at sarcopenic level; sarcopenia-L3/PM alone, only one of SMIs at sarcopenic level; and sarcopenia-both, both L3- and PM-SMIs at sarcopenic level [23]. GNRI was estimated using the following formula: 1.489 × serum albumin level (g/L) + 41.7 × [actual body weight (ABW)/ideal body weight (IBW) (kg)]. If the ABW was higher than the IBW, the ABW/IBW ratio was set to 1. According to previous criteria, GNRI scores > 98, 92 to 98, 82 to < 92 and < 82 were classified as no, low, moderate, and major risk, respectively [18].

### Statistical analysis

All analyses were performed with STATA, version 16.0 (College Station, TX, USA). Mann-Whitney U test and Chi-square or Fisher’s exact test were used to compare continuous and categorical variables between two groups, respectively. Progression-free survival (PFS) was calculated as the time from the date of R-CHOP treatment initiation to the date of progression, death, or last follow-up. Overall survival (OS) was calculated as the time from the date of R-CHOP treatment initiation to the date of death or last follow-up. Survival was plotted using the Kaplan-Meier method and compared by the log-rank test. Cox regression analysis was performed to assess the influence of clinical variables on PFS and OS. Demographics, NCCN-IPI, and other conventional prognostic factors such as B-symptoms [24], bulky disease [25], and BMI [26] were included on univariate analyses. Each factor of NCCN-IPI such as age, lactate dehydrogenase (LDH) level, Ann Arbor stage, extranodal disease, and Eastern Cooperative Oncology Group performance status (ECOG PS) was not separately analyzed to avoid multicollinearity problem. Then, all statistically significant variables with *p*-value < 0.05 on univariate analysis were included without variable selection technique in the multivariate Cox regression model. To compare the predictive performance of the models for OS, C-index, Akaike information criterion (AIC), and Bayesian information criterion (BIC) were calculated. A two-sided *p*-value < 0.05 was considered statistically significant.

## Results

### Patient characteristics

**Fig. 1:**
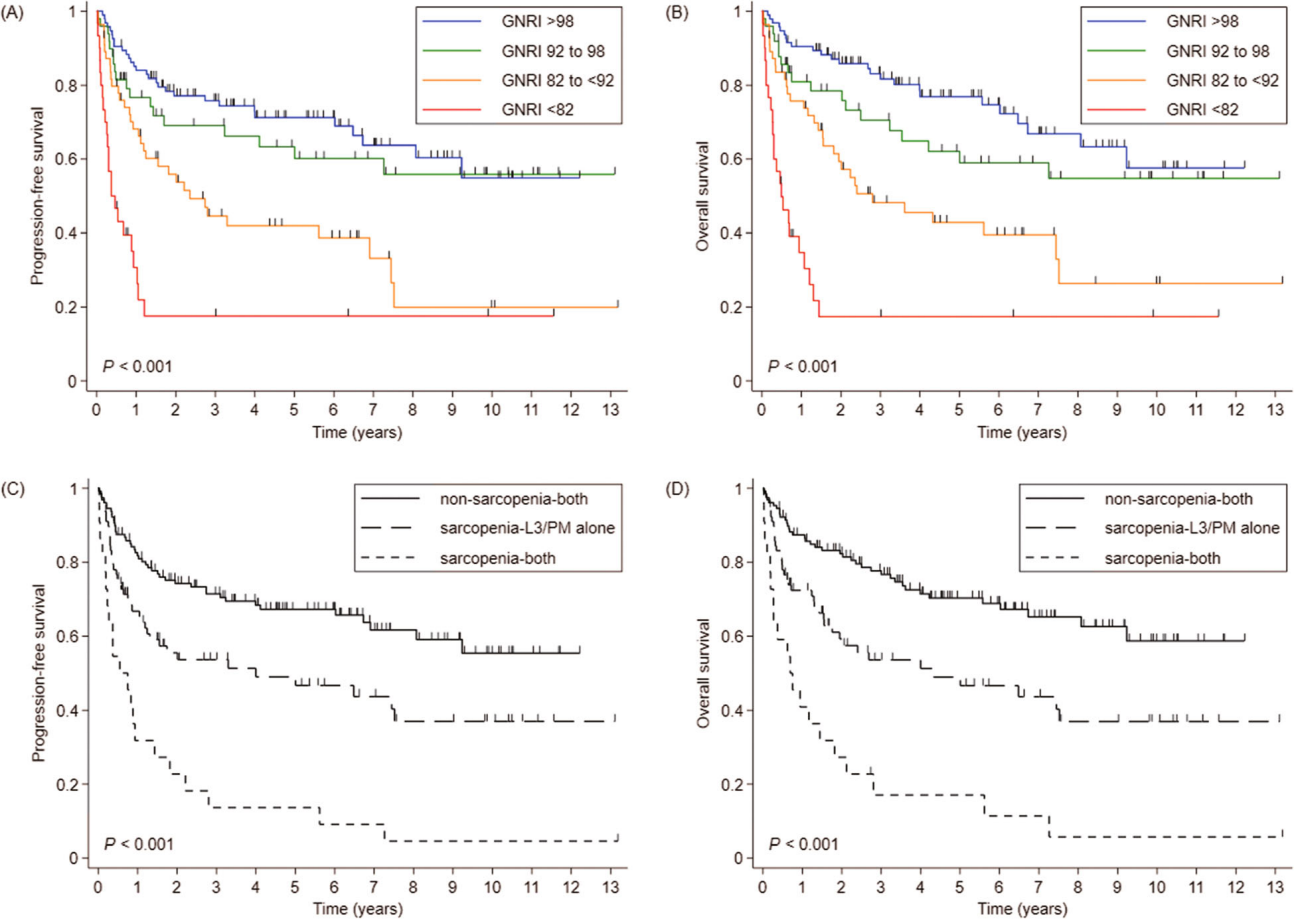
**a** Progression-free survival (PFS) and (**b**) overall survival (OS) according to the GNRI. **c** PFS and (D) OS according to the severity of sarcopenia. *Abbreviations: GNRI Geriatric Nutritional Risk Index*

**Fig. 2:**
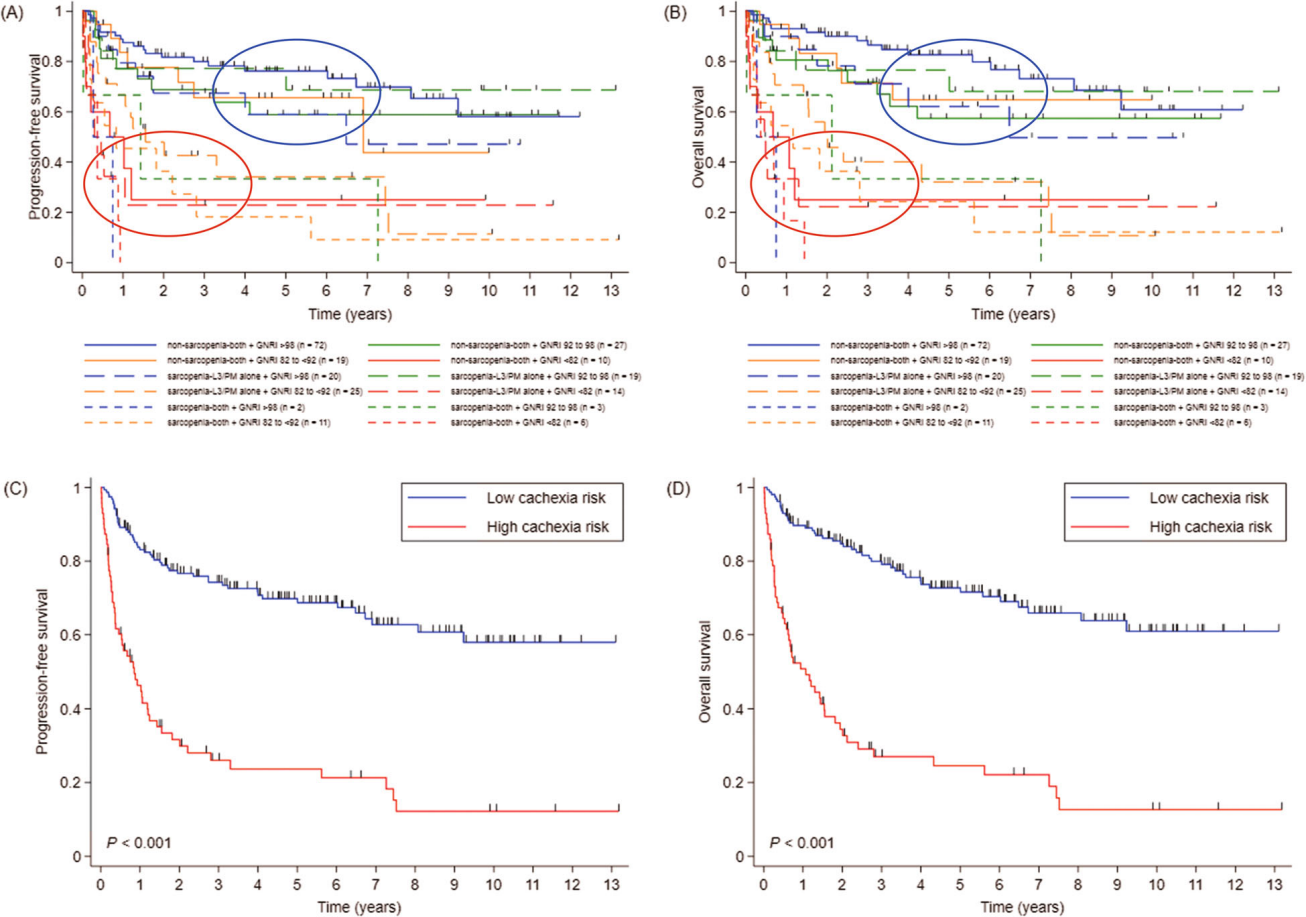
**a** Progression-free survival (PFS) and (**b**) overall survival (OS) according to the GNRI and severity of sarcopenia. Blue and red circles indicate the groups stratified into low and high cachexia risk, respectively. (**c**) PFS and (D) OS according to cachexia risk. *Abbreviations: GNRI Geriatric Nutritional Risk Index*

According to the GNRI score, 94, 49, 55, and, 30 patients were classified as no, low, moderate, and major risk groups, respectively. In terms of sarcopenia, 128, 78, and 22 patients were indicated as non-sarcopenia-both, sarcopenia-L3/PM alone, and sarcopenia-both groups, respectively. The mean (± SD) GNRIs were 97.4 (± 8.5), 91.5 (± 10.2), and 83.3 (± 10.0) in non-sarcopenia-both, sarcopenia-L3/PM alone, and sarcopenia-both groups, respectively (*p* <  0.001). PFS and OS were superior in patients with lower GNRI (Fig. 1a, b) as well as in more sarcopenic patients (Fig. 1c, d). When the survival curves were plotted against the combination of GNRI score and sarcopenic status (Fig. 2a, b), two groups emerged who exhibited significant differences in prognosis. These groups were defined as either the high cachexia risk group (HCR; *n* = 71, major GNRI risk, sarcopenia-both, or moderate GNRI risk with sarcopenia-L3/PM alone) and low cachexia risk group (LCR; *n* = 157, others).

The baseline characteristics according to the cachexia risk are listed in Table **1**. The median age was 64 years (range, 21–88 years), with 132 patients (57.9%) > 60 years old. The majority of patients had a good performance status (ECOG PS 0–1, 71.9%). There were remarkable differences in the baseline characteristics between two groups. The HCR group was associated with adverse clinical features including older age, poor PS, B-symptoms, bulky disease, advanced stage, extranodal disease, elevated LDH level, and higher IPI and NCCN-IPI. The GCB type was observed in 26 of 135 (19.3%) available patients without significant differences between groups. The BMI was lower in the HCR group relative to the LCR group (median BMI, 21.7 vs. 23.9 kg/m^2^, *p* <  0.001).

**Table 1:** Baseline characteristics

	GNRI/sarcopenia risk		*P*
	High cachexia risk (*n* = 71)	Low cachexia risk (*n* = 157)	
Median age (range), years	70 (27–88)	59 (21–86)	< 0.001
≤ 60	13 (18.3)	83 (52.9)	< 0.001
> 60	58 (81.7)	74 (47.1)	
Sex			0.111
Male	46 (64.8)	84 (53.5)	
Female	25 (35.2)	73 (46.5)	
ECOG PS			< 0.001
0–1	33 (46.5)	131 (83.4)	
2–3	38 (53.5)	26 (16.6)	
B-symptoms			< 0.001
Absent	46 (64.8)	138 (87.9)	
Present	25 (35.2)	19 (12.1)	
Bulky disease			0.009
Non-bulky	52 (73.2)	137 (87.3)	
Bulky	19 (26.8)	20 (12.7)	
Ann Arbor stage			< 0.001
I – II	17 (23.9)	83 (52.9)	
III – IV	54 (76.1)	74 (47.1)	
Extranodal disease			0.007
Absent	17 (23.9)	67 (42.7)	
Present	54 (76.1)	90 (57.3)	
LDH			0.005
Normal	19 (26.8)	73 (46.5)	
Elevated	52 (73.2)	84 (53.5)	
IPI			< 0.001
Low to Low-intermediate	19 (26.8)	107 (68.2)	
High-intermediate to High	52 (73.2)	50 (31.9)	
NCCN-IPI			< 0.001
Low to Low-intermediate	11 (15.5)	95 (60.5)	
High-intermediate to High	60 (84.5)	62 (39.5)	
Cell-of-origin (*n* = 135)			0.421
GCB	10 (23.3)	16 (17.4)	
Non-GCB	33 (76.7)	76 (82.6)	
Median BMI (range), kg/m^2^	21.7 (15.6–29.8)	23.9 (15.1–33.7)	< 0.001

Data are presented as number of patients (%) except median age and BMI

Abbreviations: *GNRI* Geriatric Nutritional Risk Index*, ECOG PS* Eastern Cooperative Oncology Group performance status*, LDH* lactate dehydrogenase*, IPI* International Prognostic Index*, NCCN-IPI* National Comprehensive Cancer Network-International Prognostic Index*, GCB* germinal center B-cell*, BMI* body mass index

### Treatment-related toxicity

Grade 3 or worse treatment-related toxicities were reported more frequently in the HCR group than in the LCR group (Table 2). The rates of grade 3 or worse anemia, febrile neutropenia, and thrombocytopenia were 31.0, 43.7, and 43.7% in the HCR group and 14.7, 26.1, and 18.5% in the LCR groups. Grade 3 or worse non-hematologic toxicities were also more common in the HCR group compared to the LCR group (49.3% vs. 30.6%). Of note, the incidence of treatment-related mortality (19.7% vs. 3.8%) and early treatment discontinuation (43.7% vs. 8.3%) was very high in the HCR group compared with the LCR group.

**Table 2:** Treatment-related toxicity

	GNRI/sarcopenia risk		*P*
	High cachexia risk (*n* = 71)	Low cachexia risk (*n* = 157)	
Hematologic toxicity, grade ≥ 3			
Anemia	22 (31.0)	23 (14.7)	0.004
Neutropenia	60 (84.5)	127 (80.9)	0.510
Febrile neutropenia	31 (43.7)	41 (26.1)	0.008
Thrombocytopenia	31 (43.7)	29 (18.5)	< 0.001
Any non-hematologic toxicity, grade ≥ 3	35 (49.3)	48 (30.6)	0.007
Treatment-related mortality	14 (19.7)	6 (3.8)	< 0.001
Early treatment discontinuation	28 (39.4)	12 (7.6)	< 0.001

Abbreviations: *GNRI* Geriatric Nutritional Risk Index

### Treatment response

In all patients, complete response (CR) was achieved in 33 of 71 patients (46.5%) with HCR and in 136 of 157 patients (86.6%) with LCR (*p* <  0.001, Table 3). CR rates of the LCR group were more than 90% regardless of the RDI of chemotherapy if the treatment was completed as scheduled. In contrast, CR rates of the HCR group were remarkably decreased, as the RDI of chemotherapy was decreased. When the treatment was prematurely discontinued, there were no statistical differences in CR rates between two groups (10.7% vs. 25.0%, *p* = 0.341).

**Table 3:** Complete response rate according to compliance for treatment

	GNRI/sarcopenia risk		*P*
	High cachexia risk	Low cachexia risk	
CR in all patients	33/71 (46.5)	136/157 (86.6)	< 0.001
CR in patients who completed treatment without DA	17/22 (77.3)	79/87 (90.8)	0.132
CR in patients who completed treatment with DA ≥ 75%^a^	11/16 (68.8)	39/42 (92.9)	0.030
CR in patients who completed treatment with DA < 75%^b^	2/5 (40.0)	15/16 (93.8)	0.028
CR in patients who early discontinued treatment	3/28 (10.7)	3/12 (25.0)	0.341

^a^Relative dose intensity of cyclophosphamide and doxorubicin ≥75%

^b^Relative dose intensity of cyclophosphamide and/or doxorubicin < 75%

Abbreviations: *GNRI* Geriatric Nutritional Risk Index*, CR* complete response*, DA* dose adjustment

### Survival

**Fig. 3:**
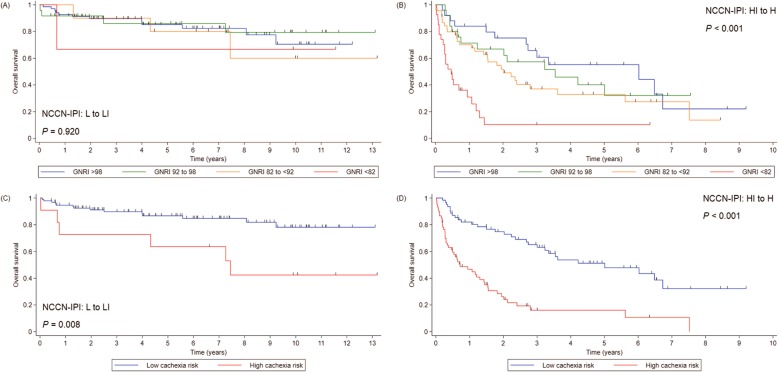
Overall survival (OS) according to the GNRI in patients with (**a**) low to low-intermediate NCCN-IPI and (**b**) high-intermediate to high NCCN-IPI. OS according to cachexia risk in patients with (**c**) low to low-intermediate NCCN-IPI and (**d**) high-intermediate to high NCCN-IPI. *Abbreviations: GNRI Geriatric Nutritional Risk Index, NCCN-IPI National Comprehensive Cancer Network-International Prognostic Index*

There were 104 PFS events and 97 deaths during the study period. With a median follow-up duration of 71.1 months, median PFS and OS of the entire cohort were 87.2 and 89.4 months, respectively. Median PFS in the HCR group was 10.3 months compared with not reached in the LCR group (*p* <  0.001; Fig. 2c). The 5-year PFS rates were 23.5 and 68.7% in the HCR and LCR groups, respectively. Median OS in the HCR group was 12.9 months and not reached in the LCR group (*p* <  0.001, Fig. 2d). The 5-year OS rates were 24.4 and 71.6% in the HCR and LCR groups, respectively. While there was no significant difference in OS according to the GNRI in the patients with low to low-intermediate NCCN-IPI, the HCR group had worse OS than the LCR group irrespective of NCCN-IPI (Fig. 3).

On multivariate analyses, the HCR group was shown to be an independent poor prognostic factor for PFS [hazard ratio (HR) 2.773, 95% confidence interval (CI) 1.826–4.212, *p* <  0.001] and OS (HR 3.348, 95% CI 2.169–5.167, *p* <  0.001) after adjusting for other covariates including the NCCN-IPI (Table 4). The predictive performance of the model for OS was best (higher C-index and lower AIC and BIC) when the cachexia risk was included in the model, instead of sarcopenia and GNRI (**Supplementary Table S****1**).

**Table 4:** Cox regression for PFS and OS

	PFS						OS					
	Univariate			Multivariate			Univariate			Multivariate		
	HR	95% CI	*P*	HR	95% CI	*P*	HR	95% CI	*P*	HR	95% CI	*P*
**GNRI/sarcopenia risk**												
Low cachexia risk	Ref.			Ref.			Ref.			Ref.		
High cachexia risk	4.308	2.915–6.367	< 0.001	2.773	1.826–4.212	< 0.001	4.961	3.302–7.452	< 0.001	3.348	2.169–5.167	< 0.001
**BMI (< 23 kg/m**^**2**^**vs. ≥ 23 kg/m**^**2**^**)**	1.016	0.691–1.494	0.935				1.096	0.735–1.632	0.653			
**NCCN-IPI**												
Low to Low-intermediate	Ref.			Ref.			Ref.			Ref.		
High-intermediate to High	5.959	3.649–9.732	< 0.001	4.342	2.580–7.308	< 0.001	6.474	3.855–10.874	< 0.001	4.793	2.770–8.292	< 0.001
**Other clinical variables**												
Sex (male vs. female)	1.121	0.757–1.659	0.569				1.109	0.739–1.664	0.619			
B-symptoms (present vs. absent)	2.574	1.694–3.913	< 0.001	1.305	0.839–2.031	0.237	2.372	1.533–3.671	< 0.001	1.173	0.742–1.856	0.494
Bulky disease (bulky vs. non-bulky)	0.874	0.513–1.490	0.621				0.810	0.459–1.428	0.466			

Abbreviations: *PFS* progression-free survival*, OS* overall survival*, HR* hazard ratio*, CI* confidence interval*, GNRI* Geriatric Nutritional Risk Index*, BMI* body mass index*, NCCN-IPI* National Comprehensive Cancer Network-International Prognostic Index

## Discussion

Our study supports the prognostic role of the GNRI in DLBCL patients. Lower GNRI was associated with worse PFS and OS. Notably, patients who did not meet any of the two criteria for sarcopenia had a favorable prognosis regardless of GNRI score, with the exception of those with major GNRI risk scores, while all patients who met both criteria for sarcopenia had an unfavorable prognosis even in cases of no GNRI risk. In contrast, for patients who met only one of the criteria for sarcopenia, disease prognoses were determined based on GNRI score. Furthermore, the predictive performance was better in the Cox model including the cachexia risk than in those including either sarcopenia or GNRI. These findings suggest that the combined use of GNRI and sarcopenia may improve the predictability of each factor in DLBCL patients.

A previous Japanese study showed that the GNRI score could identify patients with poorer prognosis among those with high-intermediate to high NCCN-IPI [19]. In contrast, a Chinese study found that while there was a marginal difference in OS by univariate analysis, GNRI score was not an independent prognostic factor for OS in multivariate analysis [20]. Given the differences in patient populations and inclusion criteria it is difficult to compare the results of our study directly with those of previous studies; however, there were considerable differences in patient characteristics between studies. The patients in the Chinese study were younger (mean age, 55 years) than those in both the Japanese study and this investigation (median ages, 68 and 64 years, respectively). The proportions of patients with low to low-intermediate NCCN-IPI were 80, 46, and 46.5% in the Chinese, Japanese, and current studies, respectively. In subgroup analyses, the GNRI score could not identify patients with a worse prognosis among those with low to low-intermediate NCCN-IPI in any these studies, whereas there was a significant association between GNRI score and OS among those with high-intermediate to high NCCN-IPI in both the Japanese and current studies. These findings may explain why the prognostic value of GNRI was differently reported in the literature [19, 20] and suggests that the GNRI alone can be a prognostic factor only in DLBCL patients with higher NCCN-IPI.

There is debate about which single parameter for cancer cachexia is most appropriate to predict the prognosis of DLBCL patients. Large database cohort studies reported that patients with low to normal BMI had shorter survival times relative to overweight or obese patients [27, 28], while subset analysis from a phase III trial failed to prove the prognostic role of BMI [29]. Sarcopenia, as determined by CT imaging, has been proposed as an independent prognostic factor in several studies [16, 23, 30, 31]. However, other studies found that the prognostic value of sarcopenia was limited in elderly and male patients [32, 33]. There are also contradictory reports regarding the prognostic role of hypoalbuminemia with various cut-off points [14, 17, 34].

Essentially, multifactorial elements are intricately linked to cancer cachexia. Muscle wasting and atrophy, which are key features in cancer cachexia, are mediated by tumor-derived factors such as proteolysis-inducing factor involving nuclear factor-κB pathway [35, 36]. Tumor-driven inflammatory cytokines are responsible for the development of cancer cachexia by inducing alterations in protein metabolism, as well as by activation of apoptosis and inhibition of regeneration of muscle mass [37]. White adipose tissue browning and lipolysis promoted by tumor-derived cytokines and hormones mediates adipose tissue and muscle wasting through molecular crosstalk between adipose and different tissues [38]. Myostatin expression and activity are enhanced in experimental cancer cachexia, with inhibition sufficient to reduce muscle loss [39, 40]. Furthermore, an international consensus has suggested that the staging criteria of cancer cachexia consist of various clinical factors, including weight loss, BMI, sarcopenia, systemic inflammation, anorexia, response to anticancer therapy, and performance status [10]. Therefore, the cachexia risk of our study, which reflects body weight, sarcopenia, and systemic inflammation may be a better surrogate marker for evaluating the severity of cancer cachexia compared with other single parameters. Cachexia risk was a predictor of treatment response, treatment-related toxicity, and survival in DLBCL. Given the intolerance to R-CHOP treatment observed in patients with high cachexia risk, dose adjustment may be considered in this group. However, chemotherapy dose adjustment resulted in a remarkable decrease of CR rate in the patients with high cachexia risk, whereas there was little effect in those with low cachexia risk. This suggests that a novel therapeutic strategy and intensive supportive care may be warranted in patients with high cachexia risk.

Our study has several limitations. First, the retrospective, non-randomized study design with a relatively small sample size makes it difficult to determine whether the differences in patients’ characteristics between the HCR and LCR groups were caused by potential selection bias or by essential differences between the two groups. In this regard, cachexia risk may be a significant confounding variable. To reduce this potential bias, all consecutive patients who were treated with the same treatment modality were included in this study. Furthermore, the prognostic value of cachexia risk was still significant after adjustment for important covariates and in stratified analysis by the NCCN-IPI. Second, laboratory biomarkers for cachexia and systemic inflammation other than serum albumin were not assessed in our study. Although serum albumin, one of the representative markers for systemic inflammation [41], was used to define cachexia risk in this study, the absence of a biomarker that better reflects the muscle wasting process may weaken the relevance of our risk model for cancer cachexia. To overcome these pitfalls, a prospectively designed study with sufficient power and sample size including various biomarkers for cancer cachexia is needed to validate our findings.

## Conclusions

Taken together, the data presented here raise the possibility of the GNRI score as a prognostic factor in DLBCL. In addition, we found that the combined risk model including GNRI and sarcopenia could better predict patient prognosis relative to GNRI alone. These findings emphasize the complexity of cancer cachexia and suggest a close relationship between cachexia, systemic inflammation, and DLBCL.

## Supplementary information

**Additional file 1: Table S1.** Comparison of predictive performance between the Cox regression models for overall survival.

## Data Availability

The dataset used and analyzed during the current study are available from the corresponding author on reasonable request.

## Abbreviations

ABC: Activated B-cell like
ABW: Actual body weight
BMI: Body mass index
CI: Confidence interval
CPA: Cyclophosphamide
CR: Complete response
DLBCL: Diffuse large B-cell lymphoma
DR: Dose reduction
DXR: Doxorubicin
ECOG PS: Eastern Cooperative Oncology Group performance status
GCB: Germinal center B-cell like
GNRI: Geriatric Nutritional Risk Index
HCR: High cachexia risk
HR: Hazard ratio
IBW: Ideal body weight
IPI: International Prognostic Index
LCR: Low cachexia risk
LDH: Lactate dehydrogenase
NCCN-IPI: National Comprehensive Cancer Network-International Prognostic Index
OS: Overall survival
PFS: Progression-free survival
PM: Pectoralis muscle
R-CHOP: Rituximab plus cyclophosphamide, doxorubicin, vincristine, and prednisone
RDI: Relative dose intensity
SMI: Skeletal muscle index
