# Supplementary material for: Prognostic model based on the geriatric nutritional risk index and sarcopenia in patients with diffuse large B-cell lymphoma
Source: BMC Cancer. 2020 May 18;20:439. doi: 10.1186/s12885-020-06921-2 (PMC7236094; doi:10.1186/s12885-020-06921-2)
Supplement: Supplementary file 1 — Additional file 1: Table S1. Comparison of predictive performance between the Cox regression models for overall survival. [file 12885_2020_6921_MOESM1_ESM.docx]

**Supplementary Table S1** Comparison of predictive performance between the Cox regression models for overall survival.

|  | C-index | AIC | BIC |
| --- | --- | --- | --- |
| Cachexia risk + NCCN-IPI + B-symptoms | 0.7664 | 871.1 | 881.4 |
| Sarcopenia + NCCN-IPI + B-symptoms | 0.7578 | 878.2 | 888.5 |
| GNRI + NCCN-IPI + B-symptoms | 0.7659 | 884.8 | 895.1 |

*Abbreviations: AIC Akaike information criterion, BIC Bayesian information criterion, NCCN-IPI National Comprehensive Cancer Network International Prognostic Index, GNRI Geriatric Nutritional Risk Index*
